# Surveillance of Foodborne Pathogens: Towards Diagnostic Metagenomics of Fecal Samples

**DOI:** 10.3390/genes9010014

**Published:** 2018-01-04

**Authors:** Sandra Christine Andersen, Jeffrey Hoorfar

**Affiliations:** National Food Institute, Technical University of Denmark, 2800 Lyngby, Denmark; sanan@food.dtu.dk

**Keywords:** culture independent, fecal sample, shotgun metagenomics, next generation sequencing

## Abstract

Diagnostic metagenomics is a rapidly evolving laboratory tool for culture-independent tracing of foodborne pathogens. The method has the potential to become a generic platform for detection of most pathogens and many sample types. Today, however, it is still at an early and experimental stage. Studies show that metagenomic methods, from sample storage and DNA extraction to library preparation and shotgun sequencing, have a great influence on data output. To construct protocols that extract the complete metagenome but with minimal bias is an ongoing challenge. Many different software strategies for data analysis are being developed, and several studies applying diagnostic metagenomics to human clinical samples have been published, detecting, and sometimes, typing bacterial infections. It is possible to obtain a draft genome of the pathogen and to develop methods that can theoretically be applied in real-time. Finally, diagnostic metagenomics can theoretically be better geared than conventional methods to detect co-infections. The present review focuses on the current state of test development, as well as practical implementation of diagnostic metagenomics to trace foodborne bacterial infections in fecal samples from animals and humans.

## 1. Why Metagenomics?

In its widest definition, metagenomics is the study of all genetic material in an environmental sample [1]. The sample can be from sea, soil, or sludge, or of human or animal origin. If the sample is from a human or animal, the focus is often to study the microbiome associated with a certain area, e.g., skin, oral cavity or intestinal tract, i.e., the collective genomes of all microorganisms found in that area [2]. In the present review, the term metagenome is used to designate all genetic material in a microbiome, especially bacteria found in the gastrointestinal tract. Due to the very limited number of animal studies, the review includes mostly references to human studies.

In the laboratory, next-generation sequencing (NGS) is the most common way to study metagenomes, either as amplicon or shotgun sequencing. The three main advantages of 16S ribosomal RNA (rRNA) amplicon sequencing, as compared to shotgun sequencing, are that (i) it is cost-effective; (ii) the data analysis can be performed by established bioinformatic pipelines; and (iii) the available reference databases are already quite comprehensive [3,4]. The two main disadvantages of 16S rRNA amplicon sequencing are that (i) the method detects a lower species richness; and (ii) it is best at classifying at the phylum, and to some extent, the genus level, whereas shotgun sequencing is better at, and below, the species level [3]. Other advantages of shotgun sequencing are that significantly more bacterial species are identified per read than with 16S rRNA amplicon sequencing, the method detects greater diversity, and it can identify organisms from other kingdoms [3]. 

An obvious area to apply metagenomics is in diagnostics. Diagnostic metagenomics has been defined by Pallen [5] as detecting and characterizing pathogens from shotgun metagenomic data. Due to its generic potential, it is ideally able to rapidly trace and diagnose all microbial pathogenic infections; bacterial, viral and parasitic, in many sample matrices; feces, urine, blood, meat, etc. The time from sample to result can be reduced to less than 24 h, compared to several days to cultivate pathogens. An excellent alternative is pathogen-specific enrichment polymerase chain reaction (PCR), though requiring separate PCR setups for each target pathogen. Together with sensitivity and specificity, which are fundamental requirements in diagnostics, the need for speeding up the time of identification (and in some cases quantification) of pathogens play a key role. However, the diagnostic metagenomics is still at an experimental stage and has not reached its full potential. 

At present, foodborne bacterial infections are diagnosed by many different laboratory methods, e.g., culturing, serotyping, resistance profiling, phage typing, hybridizations, and PCR. These methods are often laborious and take some days before a complete characterization is available [6]. It should be noted that if the method is based on culturing, it will not be able to detect infections with viable but non-culturable (VBNC) bacteria, and test samples must be grown in different culture media and under different incubation conditions to ensure detection of both aerobic, as well as anaerobic bacteria. A major disadvantage of conventional diagnostics is that the test setup is targeted, i.e., it will only find what it is designed to search for. Additionally, the success rate can be very low; a study by Vernacchio et al. [7] identified that in up to 80% of human diarrheal stool samples it was not possible to find the causative agent by conventional diagnostics. The correct treatment and patient care depend on the infecting pathogen; thus, reducing the analysis time is valuable [8,9,10]. In such cases, diagnostic metagenomics can provide a one-shot result. 

The present review focuses on the current state of test development, as well as practical implementation of diagnostic metagenomics to trace foodborne bacterial infections in fecal samples from animals and humans. First, the methods available for data analysis will be described. Subsequently, the laboratory steps necessary to include in a study are reviewed. Finally, some relevant studies are described. It should be noted that metagenomics of food samples are not included in this review due to the normally very low concentration of foodborne pathogens in such samples that often require a (pre)-enrichment step. 

## 2. The Struggle with Sequence Data Analysis

Data analysis is an important, but also difficult and time consuming, part of metagenomic studies. In practice, it can be summarized as classifying metagenomic sequences, either reads or assemblies. Classification of metagenomic sequences can be taxonomy-dependent or taxonomy-independent. The first approach, taxonomy-dependent classification, is dependent on a reference database and a comparison method, and it can be based on all sequencing data or only the marker genes [4,11]. This approach has been used in different classifiers, e.g., Kraken, published in 2014 [12], CLARK (CLAssifier based on Reduced K-mers) published in 2015 [13], Kaiju published in 2016 [14], and metagenomic mapper (MGmapper) published in 2017 [15]. An example of a reference-based tool that only focuses on a defined set of strain-specific marker genes is the metagenomic phylogenetic analysis (MetaPhlAn) [16]. All these classifiers potentially provide less information about the sequences, in return making the search against the database much faster [17]. The taxonomy-dependent methods have been first described by Mande et al. [18], while their performance has been tested by Lindgreen et al. [11]. A dedicated bioinformatics pipeline for diagnostic metagenomics, sequence-based ultra-rapid pathogen identification (SURPI), was published by Naccache et al., in 2017 [19]. It is a reference-based pipeline that uses, in a comprehensive mode, the national center for biotechnology information (NCBI) nucleotide database and the RefSeq non-redundant proteins database.

Taxonomy-dependent classification methods are more often used in diagnostic metagenomics, due to the importance for the treatment of a patient with an unknown causative pathogen. This makes databases an integrated and important part of diagnostic metagenomics. However, most available databases at present are insufficient and/or biased to include more model organisms and human pathogens [17], which in itself increases the risk of false positives, in which non-pathogens with sequence similarity to pathogens are classified as pathogens, mainly due to the absence of the correct reference [3,4]. Several dedicated databases and networks for sharing information on foodborne outbreaks are currently being developed, e.g., GenomeTrakr developed by the U.S. Food and Drug Administration [20] and the European Commission funded collaborative management platform for detection and analyses of (re-) emerging and foodborne outbreaks (COMPARE) project [21].

The second approach, taxonomy-independent classification, also called binning, is only dependent on the data itself and can be based on sequence composition, differential abundance, or both [4,22]. This approach was used in metagenomic studies, e.g., by Albertsen et al., in 2013 [23], by Nielsen et al., in 2014 [24], and by Cleary in 2015 [25]. Also, the available programs clustering contigs with coverage and composition (CONCOCT) published in 2014 [26] and MyCC (MyCambridge College) published in 2016 [27] use this approach. However, since these studies and programs do not have a diagnostic focus, and have recently been reviewed by Sangwan et al. [22], they will not be reviewed here. 

To make the classification of metagenomic sequences as correct as possible, a preprocessing of the shotgun data is often necessary. Preprocessing may include trimming, masking, and assembly. Assembly of reads into contigs improves the analysis, but is often a difficult task from a bioinformatics point of view [3,4,22]. It is, thus, recommended to examine the coverage of the metagenomic data set, as a higher coverage makes assembly and detection of differentially abundant genes more efficient [28]. Yun et al. [29] compared the two preprocessing methods trimming and masking, recommending masking over trimming, because masking showed a better false-positive rate in single nucleotide polymorphism calling. In masking, low quality bases throughout the sequence are substituted with Ns (not-detected). In trimming, which is the more frequently used of the two methods, low quality bases are removed, often only from the ends of a read, resulting in a shorter read. Segata et al. [17] recently reviewed many of the tools available for the steps in end-to-end metagenomic data analysis including preprocessing. 

Andersen et al. [30] showed the importance of knowing the pitfalls in the software used for analysis. Data from fecal samples spiked with *Campylobacter jejuni* in 10-fold dilutions were analyzed by the taxonomic classifiers Kraken and CLARK. Both classifiers identified false positive reads from *Campylobacter* in non-spiked, quantitative polymerase chain reaction (qPCR) negative samples. A sorting of Kraken hits to remove false positives was developed in that study. Briefly, the sorting was done by assigning each hit a score, keeping the high-scoring hits, and from these, removing hits for phage and plasmid DNA using the Kraken and basic local alignment search tool (BLAST) [30]. The study also illustrated a non-linear correlation between the spiking levels and the hits from metagenomic data. 

## 3. Too Much or Too Little

One of the challenges of metagenomics is to predict the presence and abundance of common, as well as rare, bacterial species: Ranjan et al. [3], who investigated the number of reads necessary to detect all abundant species, defined a species with a relative abundance of >1%, and found that it can be done with as few as 500 Illumina reads (Illumina, San Diego, CA, USA) Conversely, the study also showed that the slope of a rarefaction curve was still positive, indicating that new species were still detected with 163.7 million reads. Finally, Illumina HiSeq (2500) reads , Illumina MiSeq reads , as well as assembled reads from both platforms were compared, and it was found that the highest species detection was for the de novo assembled MiSeq reads. The study hypothesized that this was due to the MiSeq reads being a bit longer, 150–300 base pairs (bp), and thus more easily assembled into longer contigs that are more easily classified, than the HiSeq reads which were only up to 100 bp long. Andersen et al., (unpublished data) [31] illustrated how the interpretation of bacterial microbiome composition is highly influenced by the choice of method and reference database. 

Statistical analyses are often used in metagenomic studies to compare relative abundances of single organisms within a sample (alpha-diversity) or diversity among samples (beta-diversity). The most widely used methods to make results comparable are normalization and rarefying. In normalization, the ratio of an organism in proportion to the library size is calculated. In rarefying, a minimum library size is defined, then libraries with fewer reads are discarded and the remaining libraries are subsampled for all libraries to have the minimum library size. However, McMurdie and Holmes [32] argue that both strategies are statistically inappropriate, and that metagenomic data should not be considered normally distributed and should therefore be modelled using an appropriate mixture model. This conclusion is supported by Jonsson et al. [33]. 

## 4. The Effect of Infection Prevalence

At present, an interesting discussion is the differences between diarrheal patient samples and asymptomatic control samples. Bacterial richness is significantly higher in asymptomatic controls than in patients with diarrhea [34], and patient samples may contain large amounts of human host DNA [35]. Higher sequencing depths can improve replicate variability but also introduce spurious operational taxonomic units (OTUs). Frickmann et al. [36] found that about 74% of apparently healthy schoolchildren in Côte d'Ivoire, Africa, were asymptomatic carriers of one or more gastrointestinal pathogens (including bacteria, protozoa, and helminths) detected by a highly sensitive PCR. The discussion is also relevant to studies using spiked samples from healthy individuals as a model for diarrhea samples, and for studies that aim at identifying a human pathogen in asymptomatic carrier animals, e.g., *Campylobacter* in chicken; the high bacterial richness in this type of sample complicates detection of the spiking organism.

Another issue also addressed in a high-prevalence setting is co-infections, found to be quite common [37]. Becker et al. [38] demonstrated using a highly sensitive PCR that pathogen infection and co-infection were found with the same frequency among patients with persistent diarrhea and asymptomatic controls, with 84% of the participants being positive for at least one pathogen, and >50% having co-infections. However, after these results were published it was questioned whether results from developing countries, in which the incidence of many gastrointestinal pathogens is completely different than in industrial countries, are comparable, and if an appropriate group of asymptomatic controls was chosen [39]. Having said that, co-infection was also reported as being common by Joensen et al. [8] in a study using Danish human diarrhea samples. 

## 5. Laboratory Methods in Diagnostic Metagenomics

Diagnostic metagenomics is still at an early stage of development, especially with regards to the choice of laboratory techniques. However, there is no doubt that the choice of laboratory methods strongly influences the result. For example, fecal samples are complex matrices containing many different assay inhibitors and a high load of microbial commensals. Several studies have shown how sample storage, e.g., freezing, influences the bacterial composition of the extracted DNA [40,41], and how DNA extraction by different commercial kits varies significantly in both DNA yield and bacterial community composition [42,43,44]. As a model to assess the level of impurities, Josefsen et al. [44] also measured the amount of PCR-amplifiable target DNA from a spiking organism, *Campylobacter jejuni*, and found great variations between the test kits, but no correlation with the total DNA yield. A well-recognized problem in choosing an appropriate method for DNA extraction is the following dilemma: some DNA extraction methods can be gentle and thereby avoid shearing of DNA from those bacteria that are easily lysed, while other DNA extraction kits can be more harsh in order ensure that DNA is extracted from all, and not just easily lysed, bacteria [45]. In a compromised protocol, extracted DNA composition may not mimic the original composition. To make the matter worse, studies indicate that laboratory reagents and kits for DNA extraction can be contaminated by DNA which may influence the results, especially when studying low abundant bacteria in metagenomic shotgun studies [46,47].

## 6. Pre-Analytical Sample Preparation: The Messy Beginning

Andersen et al. [48] investigated a number of critical parameters in sample preparation as the indicators of sample quality and suitability for sequencing: DNA concentration, amount of qPCR-amplifiable target DNA, DNA fragmentation during library preparation, amount of DNA available for sequencing, amount of sequencing data, distribution of data between samples in a batch, and data insert size. The study found that none of the parameters showed any correlation with the target ratio of the spiking organism detected in sequencing data. However, the possible influence of the aforementioned parameters on sequencing results remains to be clarified, and the identity of other critical parameters that were not included in the study remains to be determined. A study of methods for DNA extraction by Knudsen and Bergmark et al. [49] evaluated seven different kits and a total of 16 protocols for DNA extraction on the three different matrices: human feces, pig feces and sewage. They aimed at finding a universal method to be used on all (fecal) samples, whereas most studies of DNA extraction are only evaluated on one matrix. They concluded that DNA quantities and qualities, as well as inferred microbiome composition, are dependent both on the matrix and the DNA extraction procedure. DNA concentrations varied greatly between matrices and extraction methods, and the abundance of taxa in the matrices was affected differently by the DNA extraction procedures. Methods without bead beating extracted fewer Gram positive bacteria, and there were no significant correlations between increased DNA concentration and increased community diversity or richness. 

## 7. Critical Steps in the Library Preparation

During the library preparation of sequencing libraries, fragmentation, size selection and PCR steps are known to introduce bias, and the choice of library preparation kit can influence the extracted community. Fragmentation can be done enzymatically or mechanically. Enzymatic fragmentation is often used because of the ease of use, although it results in a relatively high span in the fragment size. Mechanical fragmentation is more precise because of the smaller span in the fragment size, but in return requires specialized and expensive laboratory equipment. For the size selection, the use of solid-phase reversible immobilization beads, also known as AMPure beads (Beckman Coulter Inc., Brea, CA, USA), is recommended over gel extraction to avoid GC-bias, though the GC-bias provides a more precise size selection. The step in library preparation that introduces the most bias is PCR amplification, which is more efficient for sequences with a balanced GC-content than for sequences that are either GC- or AT-rich. PCR-free library preparation kits are recommended and are available from Illumina (TruSeq), but in return they require large quantities of input DNA (>1 µg). To minimize the GC-bias, Kapa HiFi polymerase is recommended rather than Phusion polymerase, which is the standard in Illumina library preparation kits. A deeper sequencing gives a higher precision in beta-diversity and, in addition, the detection of more OTUs [50,51,52].

The most widely used sequencers for metagenomic studies are Illumina HiSeq and MiSeq. Illumina sequencing errors do not randomly occur over the forward and reverse reads, and not all bases are equally often substituted, inserted or deleted. Most of the errors are caught by the quality scores, but some remain and can affect the result of metagenomic studies of very rare bacteria [53]. A known problem with the Illumina sequencers is carry-over of DNA in low concentrations between sequencing runs [54]. This can mask signals from rare bacteria or induce false positive hits. The carry-over can be minimized by choosing different indexes for successive runs and by running an extended washing cycle in the machine before the metagenomic sequencing runs. 

## 8. The Results so Far

Metagenomics improve foodborne diagnostics, but we need references. Although the number of studies available is limited, they all indicate the relevance and the diagnostic potential of the method. Taxonomy-dependent methods, as opposed to taxonomy-independent methods are often used in these studies because of the necessity to identify the pathogen. It is often reported that only 1% of the sequencing data are from the target pathogen [8]. The task is then to extract that 1% and identify the pathogen. This is why we still need to couple metagenomics with amplicon-based tests or another conventional tool. 

One of the first studies to apply diagnostic metagenomics on clinical fecal samples was published in 2008 by Nakamura et al. [55]. The study included two samples from one individual: one sampled during illness (illness sample) and one sampled after three months recovery (recovery sample). The data were analyzed by nucleotide basic local alignment search tool (BLASTn) of all reads against a reference database. The pathogen was identified to be *C. jejuni* because only the illness sample contained reads aligning to *C. jejuni*. Although the pathogen could be detected in metagenomic data, it was a very limited study, and besides, the dependency of the recovery sample makes the method inapplicable to real-time surveillance situations. 

Loman et al. [56] performed a retrospective analysis of 45 human fecal samples from an outbreak of Shiga-toxigenic *Escherichia coli* O104:H4 in Germany in 2011. Of the samples in the study, 40 were known to contain the pathogen. On an HiSeq (Illumina), 48 samples were paired-end sequenced with 2 × 151 bp yielding a total of 180 giga base pairs. It was possible to recover a draft genome of the outbreak strain with a coverage of >10× from 10 samples and >1× from 26 samples. Additionally, Shiga-toxin genes were identified in 27 samples. The study illustrates the potential of diagnostic metagenomics in an outbreak, but the method is not as sensitive as the conventional method used for comparison.

In 2016, Schneeberger et al. [37] published a proof-of-concept-study of diagnostic metagenomics, in which they analyzed four fecal samples from patients with persistent diarrhea using shotgun sequencing. The patients were living in an area with a high prevalence of gastrointestinal infections, also common in asymptomatic carriers and co-infections. The study included bacterial, viral and parasitic infections. The data were compared by BLASTn against the three reference NCBI databases: nucleotide, genome-specific markers (GSMer), and the comprehensive antibiotic resistance database (CARD). Each patient was positive for 8–11 different pathogens, many more than diagnosed by the conventional methods of microscopy, cultivation and multiplex PCR. One may question how many of the pathogens detected were actually causing the infection and how many were carried asymptomatically, or if some of the pathogens detected were in fact false positive hits. Nonetheless, the study demonstrated the ability to detect pathogens and co-infections from multiple kingdoms by applying a taxonomy-dependent method using the entire sequence, as well as markers and antibiotic resistance genes. 

In a study published in 2017 by Joensen et al. [8], 55 clinical fecal samples and 10 healthy control samples were analyzed using the software KmerFinder [57,58] and MGmapper [15]. The relative abundance of an organism was calculated as the percentage of the total reads in the sample assigned to that organism. The relative abundance of the pathogens investigated was compared between the three groups: (1) patients conventionally diagnosed with the pathogen; (2) patients with other infections or no conventional diagnosis; and (3) healthy controls. A sample was considered as NGS-positive if the relative abundance of a pathogen was above a threshold defined from the abundance of the pathogen in group 2 and 3, or if the sample contained pathogen-specific virulence factors. The NGS-based method identified the same pathogen as the conventional method in 34 out of the 38 samples containing bacterial pathogens. Using the NGS-approach it was surprisingly possible to identify a pathogen in five out of 11 patient samples that were in fact negative by the conventional methods. Thus, the NGS-method identified more samples with multiple infections than the conventional method. The abundance-based threshold was sufficient for identification of most of the pathogens included, but for some, e.g., *E. coli*, which is also a commensal, it was necessary to search for the pathogen-specific virulence factors. This method is actually the first one that theoretically could be applied in real-time and, interestingly, without any need for known positive or negative reference samples. However, this would require that the abundance-based threshold is calculated based on a much larger set of samples.

Another study from 2017 [35] conducted on 11 patient diarrheal samples from two foodborne outbreaks were classified by culturing (*n* = 5) and whole genome sequencing (WGS) (*n* = 6) as infections with two different strains of *Salmonella* Heidelberg. Metagenomic datasets comprising 0.06–5.5 Gigabases (GB) of microbial sequencing data were de novo assembled, and the genomes from WGS of the outbreak isolates were used as reference for extracting contigs from the pathogens by BLAST. The metagenomic results were consistent with those from culturing and WGS and it was possible to type the infection strains from the metagenomic dataset. This study proves that a metagenomic sample contains enough sequence data from a pathogen to type it, and additionally, distinguish between two outbreaks with different strains of the same species. However, as this was done using the outbreak strains as references, the difficult part will be to extract the same amount of data from the pathogen in real-time and without this very specific reference. 

In a third study published in 2017 [30], seven clinical diarrhea samples conventionally positive for *C. jejuni* with estimated infection loads from 9.2 × 10^4^–1.0 × 10^9^ colony forming units (CFU)/mL were analyzed. The study detected *Campylobacter* in all clinical samples by Kraken followed by a filtering of hits to remove false positives. The results were in line with the detection limits defined in the study from spiked human fecal samples (7.75 × 10^4^ CFU/mL). The study presents a method that could theoretically be applied in real-time situations, because it does not depend on known positive or negative reference samples. However, this would require that the method be validated on a much larger set of known samples to ensure that the filtering method is robust enough and that the hits are never false positives. The detection limits obtained in the study showed that the sensitivity of the method is comparable to that of conventional methods, although illustrating that the method, as well as the conventional methods, cannot detect low infection loads. 

## 9. What Is Next

Due to the limited number of metagenomic studies in foodborne pathogen detection, it is not possible to draw clear conclusions. However, there appear six potential strengths from the studies of diagnostic metagenomics used on human clinical samples: (1) As a proof-of-concept, pathogen reads can be identified from a patient sample when using a healthy sample from the same patient as the control; (2) Relative abundance of sequences matching the pathogen can be used to define true positive hits; (3) It can be useful and, even, necessary to search for pathogen-specific marker genes; (4) A draft genome of the pathogen can be extracted from outbreak samples; (5) A metagenome can contain enough sequence from the pathogen to type it to the strain level, and finally (6) Diagnostic metagenomics can be superior to the conventional methods when diagnosing co-infections.

Considering sequence data analysis, there has been much progress in recent years. Many new software tools and pipelines are being continuously developed, as well as new databases and platforms for sharing of data. In all the studies of human clinical samples, a reference strain or a database is used. However, future studies should consider applying a taxonomy-independent classification method. A large fraction of shotgun metagenomic data, often around 50%, is not classified due to missing reference matches. This may be overcome by the use of taxonomy-independent classification methods, depending on the fraction of data possible to assign to an OTU. In spite of this, it would still be necessary to classify these new organisms to enable a comparison of the results from various laboratories. A final consideration on shifting to the taxonomy-independent methods is, whether isolates of outbreak strains are still needed as references, or if it is enough to have a draft genome extracted from shotgun metagenomic data.

Diagnostic metagenomics has the potential for earlier and faster identification of samples with antimicrobial resistance genes, which has not been the focus of the present review but deserves a separate effort due to the clinical importance of the issue. 

## 10. Standards for Diagnostic Metagenomics

There is an urgent need for developing standards throughout the method pipeline. To this end, an important issue in the data analysis is how to identify false positives and false negatives and how to handle them. In several of the clinical studies mentioned, both false positives and false negatives were observed. This knowledge was based on a known reference sample, either from the same patient, from healthy controls or from non-spiked samples. This is a major challenge to overcome before the methods can be used in real-time diagnostics. For diagnostic metagenomics to become truly useful, the method must be able to analyze each sample in an outbreak rapidly and independently of other samples, and to provide a robust and reproducible result. 

On the laboratory side, it is necessary to develop internationally agreed, standard operating procedures for DNA extraction that ensure a correct representation of the actual microbial community. It can be discussed if the method should be focused on extracting DNA from certain organisms, e.g., bacteria or virus. By sequencing “target DNA” rather than host DNA, the sequencing capacity could be utilized more efficiently. In the current state, all steps in the laboratory, from sample storage and DNA extraction to library preparation and shotgun sequencing, are known to influence the sequence data output. However, not all of the details about the bias that can be introduced in each step are known, thus optimization is still necessary and ongoing. Ideally, diagnostic metagenomics should feature a universal method to be used on all kinds of samples with a consistent, high-quality result. Currently, this may be difficult to achieve when apparently similar (faeces) matrices show different results [48,49].

Contributing to the harmonization of DNA purification steps, a recent and important study reported a large inter-laboratory comparison of 21 DNA extraction protocols on the same fecal samples, in which differences in microbial community composition were studied [59]. It concluded that DNA extraction had the largest effect on the test outcome. 

While sharing of data, preferably raw sequencing data and associated metadata, should be the ultimate diagnostic aim in tracking of foodborne infections [21], there are still technical, methodological, legal, and, none the least, ethical barriers to overcome. Practical barriers can be standardization of DNA extraction, sequencing methods and requirements for data quality. Among parameters to be standardized is sequencing data per sample, as this has a high influence on the probability of detecting pathogens in metagenomic data. This standardization would be a balance between the analysis cost and the amount of data needed. This will hopefully be resolved with the expected decreased cost of DNA sequencing. 
